# Connexin 43 Mutations Lead to Increased Hemichannel Functionality in Skin Disease

**DOI:** 10.3390/ijms20246186

**Published:** 2019-12-07

**Authors:** Anthony G. Cocozzelli, Thomas W. White

**Affiliations:** Department of Physiology and Biophysics, Stony Brook University School of Medicine, Stony Brook, NY 11794-8661, USA; anthony.cocozzelli@stonybrook.edu

**Keywords:** connexin, gap junction, hemichannel, epidermis, disease

## Abstract

Gap junctional channels are specialized components of the cellular membrane that allow the intercellular passage of small metabolites, ions, and second messengers to maintain homeostasis. They are comprised of members of the connexin gene family that encode a wide array of proteins that are expressed in nearly every tissue type. Cx43 is perceived to be the most broadly expressed connexin in humans, with several genetic skin diseases being linked to Cx43 mutations specifically. These mutations, in large, produce a gain of functional hemichannels that contribute to the phenotypes of Erythrokeratoderma Variabilis et Progressiva (EKVP), Palmoplantar Keratodemra Congenital Alopecia-1 (PPKCA1), and others that produce large conductance and increased permselectivity in otherwise quiescent structures. Gaining functional hemichannels can have adverse effects in the skin, inducing apoptosis via Ca^2+^ overload or increased ATP permeability. Here, we review the link between Cx43 and skin disease. We aim to provide insight into the mechanisms regulating the normal and pathophysiological gating of these essential proteins, as well as address current therapeutic strategies. We also demonstrate that transient transfection of neuro-2a (N2a) cells with mutant Cx43 cDNA resulted in increased hemichannel activity compared to wild-type Cx43 and untransfected cells, which is consistent with other studies in the current literature.

## 1. Introduction

Intercellular communication is critical for cellular and tissue homeostasis within multicellular organisms, allowing for the transport of signaling molecules. Gap junctions are a key determinant in facilitating this process by allowing a direct exchange of small metabolites, second messengers, and ions [1]. In non-excitable tissue, gap junctions are involved in many physiological events, including cell differentiation, synchronization, and immune response [1,2,3,4]. This specialized intercellular coupling can also be found in excitable tissue, such as cardiac myocytes, where processes including rapid synchronization and facilitation of action potential propagation occur [5]. It is commonly known that gap junction channels are the only intercellular communication pathways that exclude the extracellular space, and are produced by the joining of adjacent connexin (Cx) channels in chordates [2,6,7,8,9].

The connexin genes form a close family displaying a large degree of amino acid identity and similarity within both the extracellular and transmembrane regions [10]. To date, the family of connexin genes is composed of 20 members within the mouse genome and 21 within the human genome, with 19 having clearly identifiable orthologs between both [11]. The genes are named starting with an abbreviation for gap junction, followed by their corresponding subgroup and order of discovery. For instance, the Cx43 gene was the first to be identified in the α-group and thus is named *GJA1*, while the Cx26 gene was the second identified gene of the β-group, and therefore is named *GJB2*. Currently, there are five recognized connexin subfamilies: *GJA, GJB, GJC, GJD,* and *GJE* [10]. The proteins are denoted according to the species from which they were derived, followed by the theoretical mass of the polypeptide, measured in kilodaltons (kDa) [12]. For instance, the approximately 32 kDa connexin protein first identified in the liver, is thus, referred to as Cx32. In order to form a complete gap junctional channel, six connexin subunits must oligomerize into a hemichannel, and then must attach to an adjacent hemichannel located on the plasma membrane of another cell. Connexins are transmembrane-spanning proteins with a half-life between one and five hours, suggesting a high turnover of gap junction channels and hemichannels per day [13,14]. Hemichannels may be formed from either single or multiple types of connexins, depending on compatibility, giving rise to heteromeric and homomeric hemichannels [15]. Connexins belonging to the same subfamily have a greater likelihood to form channels, such as A-connexins 40 and 43, rather than to those belonging to other groups [6].

Inflammation is an intricate process that serves to protect an organism against exogenous pathogens and the effects of cell damage. The inflammatory response entails a myriad of physiological events, including the recruitment of immune cells, vasodilation, increased membrane permeability, and generation of inflammatory signaling molecules [16]. Studies have shown that connexins play a key role in mediating inflammation. For example, inflammation by polycyclic aromatic hydrocarbons in lung and liver epithelial cells results in the inhibition of gap junction intercellular communication through production of arachidonic acid, chemokines, TNF, and Cox-2 activation [17,18,19,20]. In activated peritoneal macrophages, inhibition Cx43 function through either pharmacologic administration or gene knockout improved survival, indicated by a reduction in cytokines during sepsis [21]. In another example, in the inflammatory demyelinating diseases of the central nervous system, mutations in several connexin genes provide evidence that connexin channels in both oligodendrocytes and astrocytes are necessary for maintaining myelin and myelinated axonal integrity of the CNS [22].

Cx43 is perceived to be the most broadly expressed connexin in humans. Extensive studies involving Cx43 have indicated that aside from its role in communication, it can also regulate gene transcription, properties of the cytoskeleton, ATP transport, cell stress, and damage [23]. As an example of its abundance, Cx43 is widely expressed in the heart and is critical for myocyte growth and function. Genetically labeled adult rat cardiomyocytes were shown to dedifferentiate, proliferate, and electrically couple with neonatal rat ventricular myocytes through Cx43 activity and Ca^2+^-signal propagation [24]. In addition, Cx43 can be involved in pathogenic and deleterious pathways. Cx43 is critical for normal electrical conduction in the heart, for example, whereby deletions of this connexin causes arrhythmias [25]. In human diabetic retinopathy, expression of this protein was observed to be downregulated, where the degree of downregulation correlated to the amount of retinal vascular cell loss [26]. In mouse bone marrow-derived dendritic cells, Cx43 expression was increased significantly after treatment with Angiotensin II (AngII), promoting atherosclerosis and atherogenesis in the absence of an AngII type I receptor blocker [27]. Dysregulated Cx43 can also facilitate melanoma metastasis and signaling between tumor cells [28]. Truncation of the Cx43 C-terminus results in accelerated wound closure in the epidermis of transgenic mice, promoting earlier proliferation and cell migration [29]. In a mouse model of ODDD, there was an observed reduction in Cx43 expression overall, with marked decreases in gap junction coupling and plaque number, indicating certain mutations in *GJA1* may be more dominant [30]. Lastly, two heterozygous Cx43 mutants found in the bladder were studied in another ODDD mouse model, where it was found that both mutations resulted in decreased Cx43 gap junctional intercellular communication and act in a dominant manner to display defects in bladder function [31]. Since connexin proteins are crucial components in an array of central cellular events, this leaves researchers with quite a few opportunities for therapeutic intervention during pathophysiological conditions.

There are several human skin diseases that are associated with mutations in Cx43 specifically. These diseases, namely Oculodentodigital Dysplasia (ODDD) and Palmoplantar Keratoderma and Congenital Alopecia-1 (PPKCA1), among others, result in a gain in the number of functioning hemichannels across the cell membrane. ODDD is a rare autosomal dominant disorder in which patients present with congenital deformities and complications of the face, eyes, teeth, and limbs [32]. Mutations in *GJA1* encoding Cx43, in this case, can produce a variety of pathophysiological conditions that ultimately lead to ODDD, including reduced gap junction channel function, altered transport, and assembly of channels, or increased hemichannel function [33]. The goal of this article is to review the current literature on Cx43 and its specific role in the skin in normal and pathophysiological conditions, as well as discuss its association in the inflammatory response. In the epidermis, Cx43 is among the most abundantly expressed connexin proteins and is also expressed at high levels in palmoplantar skin along with Cx26. Given its ubiquitous nature, understanding its mechanism of action in each tissue, determining the level of co-expression with other connexins, and implementing therapeutic strategies for patients with these inflammatory diseases provides an ongoing challenge to current research.

## 2. Connexins and the Epidermis

### 2.1. Gating Properties of Connexins

Connexins become hemichannels following oligomerization in the ER-Golgi pathway, within either the intermediate compartment or Golgi apparatus. Once they form oligomers, hemichannels are brought to the plasma membrane to allow docking with appositional channels through their extracellular loops. Generally, it is perceived that hemichannels are delivered to the periphery of large docking areas, known as plaques, where older gap junctions are degraded and removed from the center through internalization before being replaced [34]. Undocked hemichannels may still activate under a number of conditions, such as mechanical stress, which may invoke different physiological responses by allowing the release of second messengers, including ATP or NAD^+^ [35,36]. Each hemichannel contains voltage gates, which adopt either a closed or open configuration depending on the relative polarity of the cell membrane. Within a gap junction, these gates can operate in series to determine whether the channel is in a fully open, residual, or closed configuration [37]. However, hemichannels are more likely to remain in a closed state in order to prevent the loss of integral metabolic and ionic components in a homeostatic environment [38]. However, closed channels may become open under specific circumstances through either electrical or chemical gating.

Gating is caused by alterations in channel structure that produce changes in channel conductance. In general, the magnitude of electrical coupling mediated by gap junctions is governed by the number of channels within the plasma membrane, their open probability, and their unitary conductance. Additionally, large plaque formations are another determinant for channel opening. In wild-type Cx43 immunofluorescence experiments, it was observed that coupling was absent in the presence of small protein aggregates on the plasma membrane. Further, it was estimated that cells with plaques comprising of 200–400 channels were typically uncoupled or coupled with few channels [39]. Due to the nature of gap junctions, the number of channels within plaques is driven by the expression, transport, and breakdown of connexins. In contrast, most hemichannels are activated by independent cell membrane depolarization events that are not associated with any other connexin channels [40,41]. Hemichannel opening is largely dependent on the concentration of divalent cations, such as Ca^2+^. In the case of hemichannels that are formed by various connexin isoforms, regulation by voltage can be significantly different depending on channel composition [42]. The interaction between membrane depolarization and hemichannel opening, thus invokes a repeated sequence of events, where increasing ion fluxes further depolarizes the resting membrane potential.

The permeability of gap junctional channels is governed by a number of factors. For example, the presence of sites along the pore of the channel allows for discrimination based on the size and charge of permeable solutes, of which Cx43 has been reported to possess [43]. In addition to these sites, solutes can be further selected by size based on the narrowest portion of the pore, as molecules small than this diameter may fully traverse the junction. Hydrogen bonding may also impact permeability, and it is considered a rate-limiting, where increasing the number of hydrogen bonds inversely affects solute permeation [44]. Due to the interactions between solutes and hydrogen bonds within the pore, solutes may need a specific conformation and orientation to pass based on enthalpic and entropic interactions [44]. Binding sites may also be present within the pore, which acts in a similar fashion [45]. Aside from structural constraints, gap junctional channels are sensitive to and strongly influenced by changes in voltage between the interior of both cells, known as the transjunctional voltage and membrane potential [3]. Depending on channel type, the configuration of the channel, as well as the amount of time spent in this configuration, can vary widely due to these two variables [45].

### 2.2. Connexins in Wound Healing

At its basic function, the skin is a specialized component of the human body that forms a protective barrier to the external environment. It is composed of a specialized stratified epithelium known as the epidermis. A key component of the epidermis are keratinocytes, which are located throughout the epithelium and can attach to the basal membrane, which serves as a boundary between the vascular dermis and avascular epidermis [46]. Epidermal keratinocytes are divided into the outer cornified layer, granular layer, spinous layer, and inner basal layer. Cells undergo differentiation and migration within the basal layer and are shed upon reaching the outer layers [47]. Cx43 is among the most comprehensively expressed connexin in the skin, particularly within keratinocytes.

There are several mechanisms through which connexins can be linked to skin disease and subsequent wound healing [48,49,50]. In the presence of cellular cues that drive gene expressions, such as assembly or turnover events, communication through connexin pathways may be upregulated or downregulated. After an injury, there is a transient downregulation of Cx43 in order to facilitate the healing process [51]. In general, wound healing involves the processes hemostasis, inflammation, proliferation, and maturation. Immediately following injury, hemostasis is initiated and involves the accumulation of clotting factors to prevent bleeding. Next, blood vessels enable the invasion of inflammatory leukocytes through dilation and increased permeability, resulting in phagocytosis of exogenous bacteria and damaged cells [52]. The restoration of the epithelium then proceeds by the release of several growth factors, such as epidermal growth factor (EGF) and fibroblast growth factor (FGF). The proliferation of new keratinocytes by epidermal or follicular stem cells also contribute to the restorative process [53]. The expression of Cx43 can vary during wound healing and has the ability to influence cell behaviors. Following an injury, Cx43 at the wound site becomes downregulated due to the increased production of cyclic adenosine monophosphate (cAMP) [54]. As a result, the cell junction becomes detached, and cytoskeleton remodeling ensues. In its active form, Akt can phosphorylate Cx43 and lead to further activation and migration of keratinocytes in the epidermis, promoting wound closure [54].

Numerous studies have reported that the function and activity of gap junctions and connexins play a role in nearly all aspects of wound healing, including inflammatory response coordination, signal transduction, wound healing and scarring. Further, altering Cx43 levels affect wound repair. In wound closure, mimetic sequences on extracellular loop one, called Gap26, and loop two, called Gap27, inhibit channel function by preventing hemichannel docking [55]. Connexin mimetic peptides (CMPs) have been developed to mimic small sequences of connexin proteins without altering gene expression, and include ACT-1, Gap26, Gap27, Gap19, and the L2 peptide [55]. It was observed that prolonged exposure to Gap26 and Gap27 enhances the rate of wound closure, reduce gap junction-mediated intercellular communication, and hemichannel activity in juvenile human keratinocytes and fibroblasts [56]. It was also observed that Gap27 upregulates genes associated with remodeling of the extracellular matrix, with amounts of collagen types I and III, transforming growth factor beta-1 (TGFß-1), and matrix metallopeptidase-2 (MMP-2), mRNA peaking seven days following injury in Cx43 deficient mouse skin [50]. Therefore, the expression and phosphorylated state of Cx43 in these layers of skin change throughout the wound repair process. After wound closure, expression levels of Cx43 return to homeostatic levels [51,57].

In general, the inflammatory pathway involves the recruitment of immune cells, namely neutrophils and macrophages, physiological events such as vasodilation, and increased production of cytokines and chemokines. Hemichannels are known to play a role in mediating inflammation. In this pathway, ATP can partake in either paracrine or autocrine signaling, being released into the extracellular compartment by damaged cells or tissue. During the immune activity, ATP is released from polymorphonuclear neutrophils (PMNs), a process governed by Cx43. ATP can interact with the P2X7 receptor, a class of purinergic receptors expressed in nearly all tissues within the human body [57]. PMNs serve as the first line of defense against bacterial infection and cell damage, utilizing this mechanism in inflammatory diseases like atherosclerosis and autoimmune diseases like systemic lupus erythematosus [58]. After being released, ATP feedback through another type of purinergic receptor, the P2X1 receptor, allows the accumulation of neutrophils to the affected site via chemotaxis [59]. In addition, ATP is part of a signaling pathway that begins the activation of macrophages. Pathogen-associated molecular patterns (PAMPs) or damage-associated molecular patterns (DAMPs) can interact with toll-like receptors (TLRs) located on the plasma membrane of macrophages. PAMPs are typically peptidoglycans for most bacteria and lipopolysaccharide (LPS) for gram-negative bacteria, while DAMPs are endogenous molecules that are released from damaged cells [57]. After action by PAMPs, TLRs subsequently facilitate signaling pathways like the transcription factor NF-κB and MAPK pathways. These pathways regulate the expression of a variety of cytokines, including IL-1ß, TNF-α, and IL-6 [60]. Gene expression may also be modified during the inflammatory process and may include the upregulation or downregulation of the connexin mRNA within cells [57].

### 2.3. Cx43 Hemichannels

Encoded by the *GJA1* gene, Cx43 is regulated both transcriptionally and post-transcriptionally. At the transcriptional level, activated protein kinase C (PKC) and estrogen can stimulate Cx43 expression through the transcription factor binding sites activator protein-1 (AP-1) and specificity protein-1 (SP1) sites [54]. Post-transcriptional regulation of Cx43 relies on the multiple phosphorylation sites located within the C-terminal to modulate subcellular localization and gap junction formation [54]. At least 19 of the 26 serine residues and two of the six tyrosine residues in the C-terminal region of Cx43 have been discovered to be sites of phosphorylation which direct the stability and functionality of the gap junctions [61]. Trafficking of the Cx43 hemichannel to the cell membrane involves the phosphorylation of S^364^/S^365^ [61]. This event causes a conformational change to generate the P2 isoform of the protein, a process that is necessary for plaque assembly. A previous epitome mapping experiment found that the elimination of these residues correlates with the configuration of the protein C-terminus within the Golgi and overall gap junction inclusion [62]. This suggests that modification at these regions is both necessary and occur after or upon Golgi exit. Casein kinase 1 (CK1) can then phosphorylate the S^325^/S^328^/S^330^ sites on Cx43 during the transition from the plasma membrane to influence channel gating and permeability [61,63]. Aside from intracellular trafficking, connexins have been found to be involved in extracellular communication though both extracellular vesicles and tunneling nanotubules (TNTs). TNTs are dynamic membrane structures responsible for the transport of an array of molecules in many cell types [64]. Interactions with connexins, in particular Cx43, aids in TNT-mediated electrical coupling a docking between adjacent cells by facilitating the formation and stabilization of the structure through N-cadherin [64]. Additionally, it has been found that Cx43 can facilitate the release of extracellular vesicle contents by assembling into functional channels at the surface of the vesicle. This is achieved by increasing the efficacy of luciferin delivery to luciferase-expressing cells [64].

Phosphorylation events also take place during channel closure and internalization. For example, phosphorylation of S^373^ by Akt takes place in response to induced disassembly, resulting in increased junction size. In Madin–Darby canine kidney cells transfected with wild-type and mutant Cx43 (K/R), which possess no lysine receptors for ubiquitination, it was observed that treatment of Akt and its stabilization following administration of protease inhibitors led to larger gap junction plaques [65]. This event also controls the ability of Cx43 to interact with ZO-1, a MAGUK protein related to tight and gap junction regulation [61]. Additionally, direct phosphorylation of S^279^/S^282^ is caused by the activation of Erk1/2 by various signals. Many studies indicate modifying this protein kinase or phosphorylation site can decrease the gap junction function. In an oral mucosa and skin wound experiment, female mice treated with an Erk1/2 inhibitor following injury resulted in increased Cx43 expression, promoting collagen III production and upregulation with scar formation [66]. Electrophysiology experiments utilizing mutant S^279^/S^282^ in a Cx43 knockout mouse cell line suggest that phosphorylation at these sites affect gap junction open probability [67]. Therefore, the rapid closing and internalization of intercellular communication by Erk and plaque size modulation by Akt are both vital to maintain gap junctional homeostasis.

Currently, there are one syndromic and three non-syndromic skin conditions specifically associated with Cx43 (Table 1). Typically, mutations in GJA1 encoding Cx43 result in a syndromic, autosomal-dominant disorder known as ODDD, which is characterized by craniofacial structure, syndactyly of fingers or toes, and in rare circumstances skin disease [68,69]. Hearing loss is also observed; however, unlike other connexin-related diseases, ODDD hearing loss is conductive rather than sensorineural [47,68]. There is a total of 73 mutations in Cx43 that have been linked to ODDD, of which 64 are autosomal dominant missense mutations [33]. Moreover, the mechanism underlying ODDD has been extensively investigated and hypothesized to vary depending on the mutation involved [33]. For example, Cx43-G138R has been documented to increase hemichannel function coupled with the loss of gap junction channel function [70,71]. In contrast, Cx43-G21R has been documented to elicit improper oligomerization during transport to the plasma membrane and compromise hemichannel docking, thus form plaques that are essentially non-functioning [72]. Of the non-syndromic conditions identified by mutations in *GJA1*, both EKVP and PPKCA1 share a hyperkeratosis phenotype, whereas ILVEN affects Blaschko’s lines (discussed further in Section 2.4). Investigations of the mechanism for EKVP involve Cx43-A44V and Cx43-E227D, which have been reported to result in hemichannel localization failure and subsequent Golgi accumulation [73]. Two new mutations have since been discovered and are attributable to EKVP as well, producing similar phenotypes in cases [74]. In PPKCA1, Cx43-G8V has been reported to increased hemichannel activity, resulting in increased basal levels of Ca^2+^ and its permeability [75]. Lastly, the mechanism behind ILVEN and its associated Cx43 mutation (Cx43-A44V) have not yet been fully described. Additionally, a recently discovered *GJA1* mutation (Cx43-G38E) results in a new non-syndromic phenotype whose mechanism is also not fully understood, known as Hypotrichosis with Keratosis Follicular and Hyperostosis. Mutations at this position produce characteristics similar to the other non-syndromic conditions stated, such as palmoplantar keratoderma and alopecia, but also hyperostosis of the skull and vertebrae [76].

### 2.4. Cx43 Mutations and Epidermal Pathophysiology

In large, connexin mutations that are associated with diseases contain either deletions or substitutions in single amino acids. Consequently, these mutations can be detrimental to the biochemical properties of these proteins, thus impacting the functionality of the channels that form [35,77]. Interestingly, mutations within this gene family can alter the characteristics of the hemichannel structure, often producing detrimental outcomes. For example, hemichannels may become constitutively active or dysregulated and can elicit a host of events that are catastrophic to the cell, including the depletion of essential molecules from the cytoplasm, plasma membrane depolarization, or osmotic pressure-induced cell lysis [78]. This process of keratinization can also be seen through interactions of other connexins with Cx43. For example, Cx26 mutants, which are directly associated with the keratitis-ichthyosis-deafness syndrome (KID) and palmoplantar keratoderma (PPK), can increase cell membrane permeability by interacting with Cx43 more effectively. This, in turn, can intensify Cx43 hemichannel activity; therefore, resulting in ATP leakage and Ca^2+^ overload [79,80].

In a previous study, we demonstrated that three Cx43 mutations linked to diseases of the epidermis, Cx43-G8V, Cx43-E227D, and Cx43-A44V, formed functioning hemichannels that mediated increased membrane current flow, suggesting a potential common feature of genetic skin disease related to this protein [69]. Currently, ten of the identified human connexin genes are linked to twenty-eight genetic diseases. Of these, eleven are skin disorders that contain overlapping phenotypes due to mutations in *GJA1, GJB2, GJB3, GJB4,* and *GJB6* [35,47]. Mutations in *GJA1,* in particular, can cause an array of genetic diseases, including skin disease [33,47]. For example, the single substitution mutation Cx43-G8V is linked to PPKCA1 [75]. In PPKCA1, patients typically present with severe thickening of the skin (keratosis) at the palms, knees, elbows, and feet, congenital hair loss (alopecia), and whitening of the nails (leukonychia) [81]. The single substitution mutations Cx43-A44V and Cx43-E227D were also found to be linked to skin disease, causing erythrokeratoderma varibilis et progressiva (EKVP) [82]. In EKVP, patients typically present with keratosis that is either widespread or localized to a small area of the body. EKVP cases can also present with other skin pathologies due to its association with other mutations in the connexin gene family. For example, it is not uncommon for patients with EKVP to also have palmoplantar keratoderma, which is predominantly attributable to mutations in *GJB2* [47,73,83]. In relation to Cx43 mutations, patients with EKVP can also present with leukonychia and darkening of the skin around the mouth [82]. Additionally, the Cx43-A44V mutation was also found in cases of inflammatory linear verrucous epidermal nevus (ILVEN) [84]. In ILVEN, patients typically present with reddened, raised areas (papules) that are located along the lines of normal skin development, called Blaschko’s lines [85].

**Figure 1:**
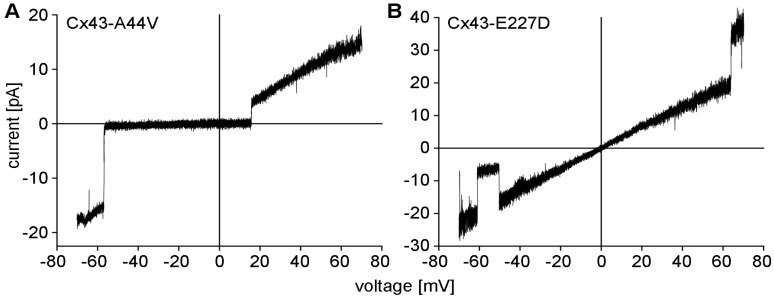
Electrophysiological data were obtained from *Xenopus* oocytes containing mutant Cx43-A44V (**A**) and Cx43-E227D (**B**) hemichannels using the cell-attached patch-clamp technique. Hemichannel responses to voltage ramps between −70 and +70 mV were recorded and plotted as unitary currents. Current-voltage relations for the Cx43 mutants were essentially linear across the tested range. Transitions to a sub-conducting state (**B**), or the fully closed state were occasionally observed (**A**).

Results from electrophysiological studies conducted in *Xenopus* oocytes that expressed mutant Cx43 cDNA showed channels with large conductances and increased function by both Cx43-A44V and Cx43-E227D (Figure 1). Under the same experimental conditions, wild-type Cx43 was unable to form these functional hemichannels [69].

**Figure 2:**
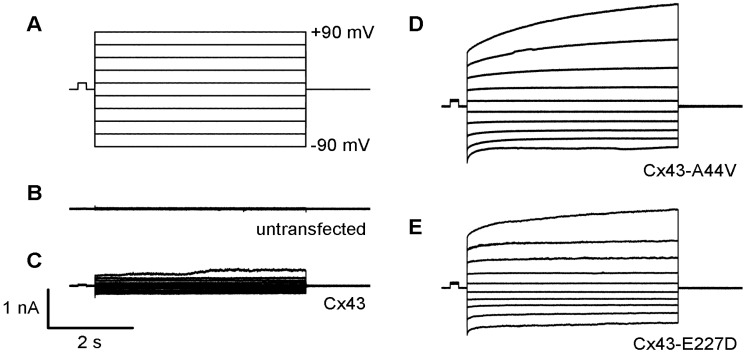
Skin disease-associated Cx43 mutations generate large hemichannel currents within transfected N2a cells (**A**). Single cells were measured using whole-cell patch-clamp technique, with a holding potential of 0 mV. Membrane current responses to voltage pulses were recorded between −90 to +90 mV at 10 mV increments. Untransfected (**B**) and wild-type Cx43 (**C**) expressing cells exhibited small membrane currents. Cx43-A44V (**D**) and Cx43-E227D (**E**) expressing N2A cells displayed larger hemichannel currents compared to the wild-type.

HeLa cells deficient in gap junctional communication were also transfected to assess mutant Cx43 function and localization. Results from immunofluorescent staining revealed proper membrane trafficking to areas of cell contact, as well as the inability of untransfected cells to form gap junctions. In contrast, the unitary conductance of transfected HeLa cells was similar to wild-type Cx43, displaying the capability to form gap junctional channels [69]. These results demonstrate that these mutations efficiently form functioning gap junctions without differences in electrophysiology, and suggest that increasing hemichannel function, evident by the mutations studied, might influence skin pathologies associated with Cx43 [69]. Consistent with these findings, transient transfection of neuro-2a (N2a) cells with Cx43-A44V and Cx43-E227D resulted in greater hemichannel activity compared to wild-type Cx43 and untransfected cells, using whole-cell patch-clamp technique (Figure 2). These results (unpublished) are also consistent with other findings in the current literature [69].

### 2.5. A Generalizable Role for Hemichannel Activity?

There are studies of other connexin mutations associated with skin pathologies that may suggest a general role for the subsequent modifications in hemichannel activity. Mutations in Cx26, for instance, are known to increase hemichannel activity and are responsible for the phenotypes observed in Keratitis-Ichthyosis-Deafness syndrome (KID) and palmoplantar keratoderma (PPK). In a KID model, syndromic mutations produced a gain of function hemichannels in HeLa cells transfected with both mutant Cx26 and Cx43, altering the oligomerization capability of both proteins and promoting intracellular Ca^2+^ and ATP release [79]. Similar results were observed in a PPK model, where there was an increased number of functioning hemichannels in *Xenopus* oocytes transfected with both mutant Cx26 and Cx43 [80]. In PPKCA1, the Cx43-G8V mutation displays large membrane currents, ion influx, particularly Ca^+2^, and cell death in comparison to wild-type Cx43 [75]. Mutations in Cx30 generate large membrane currents and increased ATP leakage in *Xenopus* oocytes as well, subsequently increasing hemichannel function [86]. Cx31 mutants associated with EKVP also demonstrate similar physiological responses, with increased activity, leakage, and cell death and damage [87]. It is crucial to note also that in each of these studies, the wild-type isoform of each connexin was less likely to form functional hemichannels, and each pathology seemed to result in connexin upregulation. As these studies suggest, a general model for an increased gain of active hemichannels may involve increases in ATP and second messenger leakage from cells, along with necrotic cell death.

Conversely, connexin skin diseases may not always be due to aberrant hemichannel activity. For example, Vohwinkel Syndrome is an autosomal dominant syndromic disease caused by mutations in the *GJB2* gene encoding Cx26 and is characterized by sensorineural deafness, palmoplantar hyperkeratosis, and pseudoainhum [88]. In a previous study, mutant Cx26 associated with Vohwinkel Syndrome (Cx26-D66H) did not form gap junctional plaques when expressed in both *Xenopus* oocytes and cervical epithelial cells and instead formed aggregates within the Golgi [89]. This failure to traffic to and assemble at the plasma membrane may be due, in part, to the inability of mutant Cx26 to interact and dock with other connexins [89]. In this case, evidence suggests a dominant-negative effect by Cx26-D66H, where a loss of function subsequently produces pathophysiological phenotypes rather than a gain-of-function described previously.

### 2.6. Current Strategies for Skin Pathology

When defective, connexins can be causal for a host of skin diseases, neuropathies, and other conditions not mentioned in this review. Given their extensive relationship, connexins can, therefore, be viable drug targets by focusing on gap junction regulation. Cx43 is an initial responder for several forms of injury, and therapeutic approaches depend heavily on disease indication and the life-cycle of Cx43 being affected [51]. For example, different approaches have been adapted to investigate the function of Cx43 in wound healing through antisense oligonucleotides and peptides in order to block channel function and interfere with Cx43 interacting proteins [55]. First, antisense oligonucleotides are short single strands of about 13–25 nucleotides that are complementary to specific mRNA. When bound to their target sequence, the expression of the target gene becomes downregulated. This type of treatment usually takes the form of a slow-releasing gel that can be applied topically to quickly downregulate the level of Cx43 in keratinocytes located at the wound edge, while decreasing the inflammatory response in blood vessels [90,91]. As mentioned previously, Cx43 antisense oligonucleotides can improve wound healing rates in a number of skin models, and therefore affect the expression of other genes involved. The reduction of Cx43 in mouse models demonstrated significantly greater rates of wound healing with thinner keratinocyte layers compared to untreated controls [92]. Second, peptides that can target the carboxyl terminus of Cx43 can be utilized to regulate channel function. For example, the A-connexin carboxyl-terminal peptide (ACT-1) can accomplish that by targeting the carboxy tail of Cx43. This prevents any interaction with ZO-1 and results in increased gap junction plaque size [46,55] by competitively inhibiting the interaction between the PDZ binding domains on both proteins [52]. Notably, ACT-1 does not interfere with expression levels, suggesting the role it plays in wound healing is not likely to be facilitated by Cx43 expression. This type of treatment also takes the form of topical applications to promote healing rates. Patients with chronic venous leg ulcers demonstrated an incidence of 100% wound closure after treatment for 12 weeks [93]. Increased rates of wound closure, diminished inflammation, and scar tissue formation were also observed in mouse and pigskin injury models, without altering Cx43 gene expression [94].

Considering normal physiological processes in humans require a sufficient level of gap junctional expression at all times, therapeutic strategies in which the expression of functional connexins is modified or regulated must proceed with caution [51]. Interventions, whether acute or chronic, have the capability of interfering with hemichannel function and gap junction coupling and produce adverse side effects. For example, it was found that expression of a Cx43 null mutation in neonatal mouse hearts resulted in further complications, such as insufficient pulmonary gas exchange, and lower rates of survival during development [95]. The use of antiarrhythmic peptides, which can reduce the effects of ischemia, cardiac arrhythmia, and atherosclerosis by modulating gap junctional communication [96], may also interfere with apoptotic cell isolation and wound healing [97]. Drug administration and targeting is a second obstacle for connexin therapeutic strategies. For example, previously developed drugs typically target wounds or disease locations that can be reached through topical applications, including skin and corneal wounds [51]. While beneficial for superficial intervention, this method can restrict systemic effects by directly applying to the wound site, and not to off-target areas in healthy tissues where connexins may also be affected [51]. Despite these limitations, therapeutic interference of connexin channels can be largely effective, given the potential to create drugs that can target both hemichannel and gap junctional isoforms with a high degree of specificity [97].

## 3. Conclusions

Connexin mutations associated with skin disease have the potential to enhance hemichannel activity in a process that may or may not affect hap junctional intercellular conductance. A gain of function mutations, such as those discussed in this review, have the capability to increase the permeability of connexin hemichannels. Our findings in electrophysiological studies revealed that while *Xenopus* oocytes transfected with Cx43-A44v, Cx43-E227D, and Cx43-G8V cDNA were still capable of forming gap junctional channels, increases in unitary conductance and transitions to sub-conducting states may be the result of a gain of functioning Cx43 hemichannels, a trend not observed in untransfected cells. Similar experiments in N2a cells also confirm this, and our findings are consistent with the current literature. Distinct roles have been identified in both pathophysiological and normal circumstances of gap junction and hemichannel function, alluding to the likelihood of the involvement of multiple mechanisms under these conditions. In the case of Cx43, such mechanisms may include increased Ca^2+^, ATP leakage, and membrane depolarization, subsequently resulting in phenotypes such as hyperkeratosis and other forms of dystrophies. However, since most of these genetic diseases are rare, whether a definitive mechanism for conditions such as PPKCA1 and ILVEN has yet to be fully investigated. Such efforts would not only improve our understanding of the role connexin hemichannels play in these disease states, but also facilitate drug targeting and increase the specificity of drugs during development.

Whether through pharmacologic or genetic models, substantial progress has been made to identify the role connexins play in the skin, and develop therapeutic strategies that target functionally active hemichannels. In recent years, utilization of connexin mimetic peptides and antisense oligonucleotides have been successful with inhibiting channel activity, directly influencing more localized regions of damage. Peptide mimetics such as ACT-1 are particularly advantageous since it can increase plaque size and restore wound healing without affecting the expression of Cx43 elsewhere in the system. While successful, therapeutics such as these still do not ensure sufficient administration of drug dosage as they are applied topically, which is a major challenge for current research. Further, these types of interventions are typically structured more towards management rather than treating disease symptoms. As a result, many questions remain concerning the intricacies of the regulatory mechanisms that direct gating and permselectivity and whether new therapeutics will have the capability of restoring epidermal homeostasis.

## Figures and Tables

**Table 1:** Connexin mutations associated with syndromic and non-syndromic skin disease.

Pathology	Connexin	Gene	Mutation	Clinical Features	Mechanism Linked to Pathology?
Erythrokeratoderma Variabilis et Progressiva (EKVP3) (OMIM 617525)	Cx43	*GJA1*	A44V (131C-T); E227D (681A-T); P283L (848C-T); T290N (869C-A)	Widespread or Localized Keratosis, Palmoplantar Keratoderma	Hemichannel Functionality or Unknown
Inflammatory Linear Verrucous Epidermal Nevus (ILVEN)	Cx43	*GJA1*	A44V (131C-T)	Raised Papules along Blaschko’s Lines	Hemichannel Functionality
Oculodentodigital Dysplasia (ODDD) (OMIM 164200)	Cx43	*GJA1*	Y17S (50A-C); S18P (52T-C); G21R (61G-A); G22E (65G-A); V96M (286G-A) ^1^	Craniofacial, Dental, Ocular, and Digital Abnormalities, Syndactyly	Hemichannel Functionality
Palmoplantar Keratoderma and Congenital Alopecia-1 (PPKCA1) (OMIM 104100)	Cx43	*GJA1*	G8V (23G-T)	Keratosis of Palms, Knees, Elbows, and Feet, Alopecia, Leukonychia	Hemichannel Functionality
Hypotrichosis with Keratosis Follicular and Hyperostosis	Cx43	*GJA1*	G38E (113G-A)	Leukonychia, Palmoplantar Keratoderma, Hyperostosis, Alopecia	Unknown

^1^ There are a total of 73 Cx43 mutations currently associated with ODDD [33].
